# Structural Equation Model on the Problem Behavior of Adolescents

**DOI:** 10.3390/ijerph20010756

**Published:** 2022-12-31

**Authors:** Eun Mi Kim, Sona Lee, Hye Young Ahn, Hye Seon Choi

**Affiliations:** 1 College of Nursing, Eulji University, Uijeongbu 11759, Republic of Korea; 2 College of Nursing, Woosuk University, Wanju 55338, Republic of Korea

**Keywords:** adolescent, child abuse, problem behavior, psychological maltreatment, self-control, resilience, psychological, social support

## Abstract

This study aimed to explain direct and indirect relationship between psychological maltreatment, socio-psychological prevention factors, and problem behavior of adolescents based upon Jessor’s protective-risk model and Haase’s adolescent resilience model (ARM). A convenience sample of 138 Korean adolescents was recruited for the cross-sectional survey design. Using the collected data, the developed model was verified by structural equation modeling analysis using SPSS and AMOS program. Regarding model fit, χ^2^ = 151.62 (*p <* 0.001), GFI = 0.908, AGFI = 0.836, CFI = 0.911, SRMR = 0.060, and RMSEA = 0.10, showing acceptable fit levels. Psychological maltreatment explained 11.5% of perceived social support; psychological maltreatment, perceived social support, and self-control explained 89.9% of resilience; psychological maltreatment and perceived social support explained 53.2% of self-control; and psychological maltreatment, perceived social support, resilience, and self-control explained 39.7% of problem behavior. Psychological maltreatment directly and indirectly influenced perceived social support, self-control, and problem behavior. Psychological maltreatment and self-control were the factors that influence problem behavior of adolescents. The findings suggest that psychological maltreatment must be eradicated to reduce problem behavior of adolescents and enhance their socio-psychological protection factors.

## 1. Introduction

Recently, interests in child abuse are increasing in Korea, as related death cases are reported [1]. Since the revision of the Child Welfare Act in 2018, there has been analysis in Korea on the current status of child abuse victims, protection and support, actual cases, policies for child abuse prevention, etc. As a result, the number of child abuse cases has continued to increase according to 2019 statistics of child abuse (Child Abuse & Neglect Korea 2019). The number of child abuse cases has increased drastically: 22,377 in 2017, 24,604 in 2018, and 30,045 in 2019 [2].

There are four types of child abuse: physical maltreatment, psychological maltreatment, sexual maltreatment, and negligence [3]. Among these, psychological maltreatment indicates rejective or indifferent attitudes of the parents or rearers and acts of threatening a child or promoting acts of deviation. Such maltreatment hinders the child’s physical psychological and social development [4]. Psychological maltreatment is one of the factors that cause problem behaviors among adolescents, affecting the physical and psychological growth of adolescents negatively [5]. Unfortunately, the number of psychological maltreatment cases was 7622, the largest among child abuse types, and the age group where child abuse occurred most frequently was adolescents aged 13 to 15, according to the 2019 annual report of child abuse by the Ministry of Health and Welfare (KOHW) [3]. While the occurrence rate of psychological maltreatment in Korea is so high, current child abuse policies focus on physical maltreatment, with the criticality of psychological maltreatment neglected [1,3].

Children and adolescents who experience psychological maltreatment suffer damage to physical and psychological development [4] and exhibit various problem behaviors such as negative self-esteem, anxiety, depression, shrinkage, aggression, etc. [6,7,8,9,10]. Adolescence is a transition period from childhood to adulthood during which drastic physical and psychological changes make adolescents feel sensitive [4]. Moreover, adolescents are psychologically quite unstable since they are easily affected by conflicts with parents or companion groups as well as changes in social environments [11]. Particularly, psychological maltreatment that adolescents experience affects their childhood negatively. Regulating child abuse for adolescents, therefore, is as important as that for children. Fortunately, not every adolescent who experiences child abuse develops problem behaviors or aggressive tendency [12]. Each adolescent’s socio-psychological factors act as a protective factor that reduces problem behaviors [13].

Three major socio-psychological protective factors that affect adolescents’ problem behaviors are social support, self-control, and resilience [9,14,15]. In adolescence, the occurrence probability of problem behaviors depends on the social support that adolescents experience. As adolescents receive positive support from persons important to them such as parents, friends, and teachers, the occurrence rate of problem behaviors decreases [10,13]. Self-control means the ability to control one’s emotions and impulses in order to achieve an important or long-term goal [7,15,16]. Self-control reduces the risk of externalizing internalizing problem behaviors among adolescents [14,17] as well as aggressive and anti-social behaviors and anger among adults [15]. Resilience is a positive psychological resource to explain a feeling of happiness among adolescents [18]. This is an ability to overcome adversities, recover from psychological conditions, and adapt oneself positively in challenging circumstances [5,19]. It is reported that resilience affects the mental condition and subjective feeling of well-being among adolescents, playing an important role in the prevention and treatment of problem behaviors and related education [19,20]. Abused adolescents showed lower resilience than non-abused adolescents. In addition, in a previous study targeting adolescents, resilience was shown to mediate between emotional abuse and problem behaviors [5,19]. Recently, resilience has been shown as a mediating variable and an important protective factor in the effects of emotional abuse on borderline personality disorder [21], and resilience was found to moderate the effects of prior childhood maltreatment on externalizing problems [22]. As such, social support, self-control, and resilience may function as protective factors to adolescents exposed to psychological maltreatment.

Previous studies on child abuse among adolescents have focused on the problem behaviors of adolescents who have experienced maltreatment. There has been little research on psychological maltreatment and adolescent problem behaviors among child abuse types. Particularly, there has been little research on modeling to explain factors affecting psychological maltreatment and adolescent problem behaviors and relationships among them. Two examples of models explaining adolescent problem behaviors are Jessor’s protective-risk model [23] and Haase’s adolescent resilience model (ARM) [24]. The protective-risk model [23] is based on the theory of problem behaviors [25]. In this model, it is explained that when the protective factors for adolescents are low, the risk factors for problem behavior have a greater effect, and when the protective factors are high, the risk factors for problem behavior decrease [23,26]. The adolescent resilience model (ARM) [24] consists of risk factors and protective factors that affect adolescents’ quality of life. This model explains that risk factors affect protective factors and resilience negatively, while protective factors affect resilience positively. It also explains the path that ultimately affects the quality of life [24]. Therefore, these two models are appropriate as a theoretical framework for modeling related to adolescents’ psychological maltreatment and problem behaviors.

Accordingly, this study examines the influence and path from psychological maltreatment to adolescents’ problem behaviors, with variables of socio-psychological protective factors (social support, self-control, and resilience) based on the theoretical framework of Jessor’s protective-risk model [23] and Haase’s adolescent resilience model (ARM) [24].

## 2. Materials and Methods

### 2.1. Study Design

In this study, a conceptual framework was constructed to build a hypothetical model composed of factors influencing adolescents’ problem behavior. After that, we built a structural equation model that selects variables, collects data cross-sectionally, and tests the effect of the hypothetical model based on the collected data.

#### Hypothetical Model

With Jessor’s protective-risk model [23] and Haase’s adolescent resilience model (ARM) [24] as the theoretical framework, a hypothetical model was established to analyze factors affecting adolescents’ problem behaviors. The protective-risk model was composed of protective factors and risk factors for adolescent problem behavior. Protective factors directly help adolescents to engage in desirable and positive behaviors and indirectly act as a buffer to reduce risk factors that can cause problem behaviors. Risk factors increase problem behaviors in adolescents. The adolescent resilience model (ARM) consists of risk factors and protective factors that affect adolescent resilience and quality of life. The adolescent resilience model (ARM) includes pathways in which risk factors negatively affect protective factors and resilience and pathways in which protective factors positively affect resilience. The results of these factors are presented as having an impact on quality of life.

**Figure 1:**
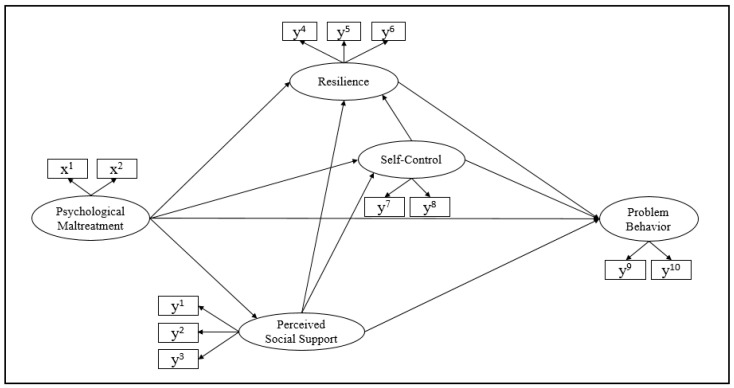
Hypothetical Model of Problem Behavior among Adolescents. x^1^, emotional abuse; x^2^, neglect; y^1^, family; y^2^, friend; y^3^, teacher; y^4^, affirmative; y^5^, controllability; y^6^, sociability; y^7^, long-term control; y^8^, short-term control; y^9^, internalizing; y^10^, externalizing.

The hypothetical model represents the effects of psychological maltreatment, perceived social support, resilience, and self-control on adolescent problem behavior. In addition, direct and indirect pathways through which psychological maltreatment affects problem behaviors of adolescents through protective factors were included. This hypothetical model has one exogenous variable (psychological maltreatment) and four endogenous variables (perceived social support, resilience, self-control, problem behavior) (Figure 1).

### 2.2. Study Participants

The subjects of this study were students enrolled in middle school or high school in K Province and G City selected through convenience sampling. We visited four schools and collected data in the classroom with permission from the teachers and students of the department. The criteria for inclusion of research subjects are students who have heard and agreed to the purpose of this study. In addition, students should be able to read and understand the contents of the questionnaire and answer the questions. Exclusion criteria are students who disagree with this study.

A total of 250 individuals was selected based on the minimal sample size (200) suggested by the maximum-likelihood classification method and the withdrawal rate according to the asymptotically distribution-free method [27]. Likewise, 250 questionnaires were distributed and collected 100%, but 12 copies with insufficient answers were excluded, and 238 copies were used in the final analysis.

### 2.3. Measurements

#### 2.3.1. Psychological Maltreatment

In order to measure psychological maltreatment perceived by adolescents in this study, the translated and revised version of the CTS (Conflict Tactics Scale) of Shin et al. [28] and Straus [29] was utilized, and this study includes a survey where 9 questions regarding the “emotional abuse” item and 10 questions regarding the “neglect” item were used. After data were collected through this tool, questions 13 and 15, whose reliability turned out to be low according to the reliability analysis, were removed, and then, the remaining 17 questions were utilized. This tool’s range of score is between 17 and 68 points. A high score indicates that the respondent has experienced more abuse. Shin et al.’s research reliability was Cronbach’s α = 0.87 [28], and this study’s reliability was Cronbach’s α = 0.65. Cronbach’s α is a reliability coefficient that provides a method of measuring internal consistency of tests and measures. Internal consistency is satisfied when Cronbach’s alpha is 0.6 or higher.

#### 2.3.2. Perceived Social Support

Perceived social support was measured by means of the social support tool [30] developed by Kim. This 5-point Likert scale includes 23 questions: 8 questions about family support, 8 questions about companion support, and 7 questions about teacher support. This tool’s range of score is between 23 and 115 points. A high score indicates that the respondent perceives his/her social support as high. Kim’s research reliability was Cronbach’s α = 0.84 [30], and this study’s reliability was Cronbach’s α = 0.94.

#### 2.3.3. Resilience

Resilience was measured by means of the resilience tool [20] developed by Shin et al. for adolescent research. This 5-point Likert scale consists of three sub-factors: controllability, positiveness, and sociability. It includes 27 questions in total. This tool’s range of score is between 27 and 135 points. A high score indicates that the resilience is high. Shin et al.’s research reliability was Cronbach’s α = 0.88 [20], and this study’s reliability was Cronbach’s α = 0.94.

#### 2.3.4. Self-Control

Self-control was measured by means of the self-control scale [31] developed by Nam et al. for adolescent research. This 5-point Likert scale consists of 20 questions in total: 10 questions about short-term control and 10 questions about long-term control. After data were collected through this tool, question 20, whose reliability turned out to be low according to the reliability analysis, was removed, and then, the remaining 19 questions were utilized. This tool’s range of score is between 19 and 95 points. A high score indicates that the self-control is high. Nam et al.’s research reliability was Cronbach’s α = 0.78 [31], and this study’s reliability was Cronbach’s α = 0. 84.

#### 2.3.5. Problem Behavior

Problem behaviors were measured by means of the adolescent self-report (K-YSR) [32] tool, which is part of the Korean translation version (Korea Child Behavior Checklist, K-CBCL) of the Child Behavior Checklist (CBCL) of Achenbach, translated and standardized by Oh et al. [33]. This tool consists of 31 questions about internalizing problem behaviors and 32 questions about externalizing problem behaviors. After data were collected through this tool, questions 1, 35, and 61, whose reliability turned out to be low according to the reliability analysis, were removed, and then, the remaining 60 questions were utilized. This tool’s range of score is between 60 and 180 points. A higher score means more problem behavior. Oh et al. study’s reliability was as follows: For internalizing problem behaviors, Cronbach’s α = 0.91, and for externalizing problem behaviors, Cronbach’s α = 0.86 [32]. This study’s reliability was as follows: For internalizing problem behaviors, Cronbach’s α = 0.91, and for externalizing problem behaviors, Cronbach’s α = 0.85.

### 2.4. Data Collection and Ethics

Before data collection for this study, the survey was approved (IRB #: **16–47) by the Institutional Review Board (IRB) of ** University to which the researcher belongs. Prior to the survey, the questionnaire was reviewed by two professors of nursing and revised in reflection of expert opinion in order for adolescents’ clear understanding. Data collection was conducted for 2 months from November to December 2016. The subjects of this study were 2nd to 3rd grade students in four middle schools and 1st to 2nd grade high school students in K Province and G City, selected through convenience sampling. For this study, the research objective and gist of the study were explained to the school principal, the teacher in charge, and health teacher at the school before the survey in order to request their cooperation.

The survey was conducted directly by the researcher, who visited each classroom. Before the questionnaire was distributed, the research objective, questionnaire contents, and privacy policy were explained to subjects. It was notified that collected data would be used for no other purpose but research. Subjects were also sufficiently informed that they could withdraw from the research. The questionnaire was distributed among subjects who fully understood the research contents, agreed to participate voluntarily, and signed the agreement. Completed questionnaires were put into an envelope and collected directly by the researcher. Before data analysis, collected data were coded for personal data protection.

### 2.5. Data Analysis

The collected data were analyzed using SPSS (IBM Corporation, New York, NY, USA) for Windows 24.0 and AMOS 24.0. Collected demographic variables of subjects were analyzed in reference to descriptive statistics such as frequency and percentage. The reliability of the scales was verified by Cronbach’s α. Measured variables were analyzed in reference to descriptive statistics such as average, standard deviation, skewness, and kurtosis. Correlations among psychological maltreatment, perceived social support, resilience, self-control, and problem behaviors were analyzed in application of Pearson’s correlation. To test the fit of the model, χ^2^ statistics, GFI (Goodness of Fit Index), AGFI (Adjusted Goodness of Fit Index), CFI (Comparative Fit Index), SRMR (Standardized Root Mean Residual), and RMSEA (Root Mean Square Error of Approximation) were used. The parameter-estimated value of measured variables was analyzed as the SRW (Standardized Regression Weight), RW (Regression Weight), CR (Critical Ratio), SMC (Squared Multiple Correlations), and SE (Standard Error). The statistical significance of the direct effect, indirect effect, and total effect was confirmed by bootstrapping.

## 3. Results

### 3.1. Participant Demographics and Characteristics

The gender ratio of subjects is 52.1% (male) to 47.9% (female). As for school types, academic high schools accounted for the largest portion (50.4%) and then middle schools (30.7%) and vocational high schools (18.9%) in order. As for companion relationships, 85.7% answered “good”. As for stress from schooling, the largest portion (65.5%) answered “normal”. As for family types, the largest portion (67.2%) answered “living with parents”. As for economic conditions (53.8%), the largest portion answered “middle”. As for the father’s academic background, the largest portion (42.4%) answered “a college graduate”. As for the mother’s academic background, the largest portion (50.0%) answered “a high school graduate”. As for the father’s vocation, the percentages were in the order of office work (37.8%), technical production (30.3%), sales service (20.2%), etc. (11.8%). As for the mother’s occupation, the percentages were in the order of “working” (64.7%) and “a housewife” (31.9%) (Table 1).

### 3.2. Research Variables’ Descriptive Statistics and Correlation

The average of emotional abuse is 9.56 ± 1.12 points, and that of negligence is 8.34 ± 0.87 points. As for perceived social support, the average of family support is 33.28 ± 6.21 points, that of companion support 30.98 ± 5.20, and that of teacher support 25.36 ± 4.72 respectively. As for resilience, the average of controllability is 31.67 ± 5.27 points, that of positiveness 33.74 ± 5.89, and that of sociability 33.78 ± 5.28, respectively. As for self-control, the average of long-term control is 33.81 ± 5.08 points, and that of short-term control is 38.02 ± 5.27 points. As for problem behaviors, the average of internalizing problem behaviors is 8.97 ± 8.34 points, and that of externalizing problem behaviors is 6.68 ± 5.72 (Table 2).

The data analysis of the structural equation model meets multivariate normality. Because of the significant difference between adolescents who experienced psychological maltreatment and those who did not, we found regarding the data that skewness and kurtosis exceeded the common level. Thus, when the absolute value of skewness exceeds 3, or the absolute value of kurtosis is 8 or larger, it is viewed as extreme kurtosis. If the absolute value of kurtosis exceeds 10, the normality is problematic. If it exceeds 20, the problem is more serious according to the criteria [34]. Among the variables measured in this study, no absolute value of skewness was 3 or larger, and no absolute value of kurtosis was 8 or larger. Thus, the assumption about normal distribution is correct.

As for perceived social support for psychological maltreatment, there was a significant negative correlation with family support (r = −0.283, *p <* 0.001), companion support (r = −0.153, *p* = 0.018), and teacher support (r = −0.170, *p* = 0.009). As for resilience, there was a significant negative correlation with controllability (r = −0.167, *p* = 0.010) and positiveness (r = −0.222, *p* = 0.001). As for self-control, there was a significant negative correlation with long-term control (r = −0.142, *p* = 0.029). As for psychological maltreatment, there was a significant static correlation between internalizing problem behaviors (r = 0.321, *p* < 0.001) and externalizing problem behaviors (r = 0.231, *p* < 0.001). As for neglect, there was a significant static correlation with family support (r = −0.181, *p* = 0.005) and internalizing problem behaviors (r = 0.200, *p* = 0.002) and externalizing problem behaviors (r = 0.189, *p* = 0.003) (Table 3).

As for data analysis for the structural equation model, if the absolute value of the coefficient of correlation between measured variables is 0.90 or larger, there can be a problem in terms of multi-collinearity [35]. In this study, the absolute value of the coefficient of correlation between measured variables was all under 0.70, and thus, there is no problem in terms of multi-collinearity.

### 3.3. Verification of the Hypothetical Model

#### 3.3.1. Fitness Verification of the Hypothetical Model

The hypothetical model of this study meets the criteria of multivariate normality. Thus, for parameter estimation, the maximum likelihood (ML) method was utilized. For parameter estimation, the factors—psychological maltreatment, companion, positiveness, long-term control, and internalizing—were fixed to 1, and the rest of the factors’ parameters were estimated. Measured variables explained the potential variables significantly (Table 4).

The following result of the hypothetical model fitness verification shows the acceptability: χ^2^ = 151.62, *p* < 0.001, GFI = 0.908, AGFI = 0.836, CFI = 0.911, SRMR = 0.060, and RMSEA = 0.10 [34,35] (Table 5). 

#### 3.3.2. Analysis of the Hypothetical Model

**Figure 2:**
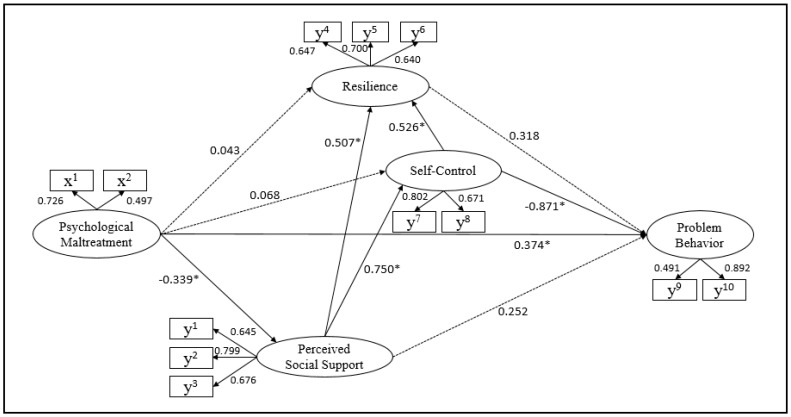
Hypothetical Path Diagram of the Hypothetical Model. x^1^, emotional abuse; x^2^, neglect; y^1^, family; y^2^, friend; y^3^, teacher; y^4^, affirmative; y^5^, controllability; y^6^, sociability; y^7^, long-term control; y^8^, short-term control; y^9^, internalizing; y^10^, externalizing. * *p <* 0.05.

The result of the hypothetical model analysis is as follows (Figure 2):

The statistically significant paths in this study’s hypothetical model were as follows: perceived social support and psychological maltreatment (γ = −0.339, *p* = 0.006), resilience and perceived social support (β = 0.507, *p* < 0.001), resilience and self-control (β = 0.526, *p* < 0.001), self-control and perceived social support (β = 0.750, *p* < 0.001), problem behaviors and psychological maltreatment (γ = 0.374, *p* = 0.012), and problem behaviors and self-control (γ = −0.871, *p* = 0.027) (Figure 2).

Psychological maltreatment explained perceived social support as much as 11.5%, while psychological maltreatment, perceived social support, and self-control explained resilience as much as 89.9%. In this study, psychological maltreatment and perceived social support explained self-control as much as 53.2%, while psychological maltreatment, perceived social support, resilience, and self-control explained problem behaviors as much as 39.7% (Table 6).

#### 3.3.3. Analysis of Hypothetical Model Effects

The direct effect, indirect effect, and total effect of factors related to psychological maltreatment and adolescents’ problem behaviors are as shown below (Table 7):

The direct effect of psychological maltreatment on perceived social support was (β = −0.339, *p* = 0.047) significant. The direct effect of perceived social support on resilience (β = 0.507, *p* = 0.023) and the direct effect of self-control on resilience (β = 0.526, *p* = 0.004) were significant. The direct effect of psychological maltreatment on self-control was not statistically significant, but its indirect effect (β = −0.254, *p* = 0.037) was significant. The direct effect of perceived social support on self-control was (β = 0.750, *p* = 0.003) significant. The total effect of psychological maltreatment on problem behaviors (β = 0.379, *p* = 0.024) and that of self-control on problem behaviors (β = −0.703, *p* = 0.043) were significant. However, the direct effect, indirect effect, and total effect of perceived social support and resilience on problem behaviors were all not significant (Table 7).

In other words, it turned out in this study that psychological maltreatment affects perceived social support, self-control, and problem behaviors directly or indirectly. In addition, it turned out that variables affecting adolescents’ problem behaviors are psychological maltreatment and self-control.

## 4. Discussion

This study examines the direct or indirect effects of psychological maltreatment, perceived social support, resilience, and self-control on adolescents’ problem behaviors based on the theoretical framework of Jessor’s protective-risk model [23] and Haase’s adolescent resilience model (ARM) [24].

The findings of this study indicate that psychological maltreatment affects adolescents’ perceived social support, self-control, and problem behaviors directly or indirectly. Psychological maltreatment, perceived social support, resilience, and self-control explained adolescents’ problem behaviors as much as 39.7%. In other words, adolescents’ problem behaviors are affected by socio-psychological factors (perceived social support, resilience, and self-control), and thus, socio-psychological factors may be utilized in strategies to reduce adolescents’ problem behaviors. In addition, psychological maltreatment explained perceived social support as much as 11.5%, while psychological maltreatment, perceived social support, and self-control explained resilience explained as much as 89.9%. In addition, psychological maltreatment and perceived social support explained self-control as much as 53.2%. As such, resilience self-control showed significant explanatory power in this study. This indicates that socio-psychological factors are of importance in managing adolescents’ problem behaviors that result from psychological maltreatment. In addition to this, an approach to enhance socio-psychological protective factors of adolescents is necessary in order to reduce such problem behaviors.

The direct and indirect effects of psychological maltreatment on adolescents’ problem behaviors were not demonstrated, but the total effect turned out to be a significant static impact. This finding corresponds to another overseas research finding that psychological maltreatment is a predictive factor of adolescents’ problem behaviors [5,36]. This finding also corresponds partially to a domestic research finding that patents’ negative rearing attitudes are related to the child’s problem [17,37]. Maltreatment affects the development of both children and adolescents negatively. Particularly in the case of children and adolescents who experience psychological maltreatment, the negative effect is long-lasting and even threatens mental health in the adulthood [7,36]. In order for healthy growth and development of children and adolescents, psychological maltreatment must therefore be eradicated. In addition, parents must play the important role of creating supportive rearing environments. In Korea, there is a cultural characteristic that psychological maltreatment is a kind of discipline [38,39]. Accordingly, the social recognition of child abuse is quite low in Korea [40]. In order to promote proper perception of child abuse, it is therefore necessary to strictly distinguish discipline from maltreatment. In addition, it is necessary to strengthen punishments regarding child abuse and to re-establish legal provisions [40].

The results showed that perceived social support had direct and significant static effects on resilience and self-control. This finding is consistent with the findings of previous studies at home and abroad that social support affects coping ability, psychological feeling of well-being, and resilience among children [41,42]. This finding also supports the theoretical framework that social support for adolescents affects individuals’ psychological protective factors positively [24]. Therefore, it is important to establish a positive relationship with the social support system, including the family, companions, and teachers, as part of strategies to reduce problem behaviors among adolescents who experience psychological maltreatment. Strengthening the social support system through positive relationships with meaningful people will be of help in improving resilience and self-control among adolescents. In this study, perceived social support proved to have no direct or indirect significant effect on problem behaviors. This is different from the finding of previous studies that social support prevented adolescents’ problem behaviors and had significantly positive effects on their growth and development [43,44]. This is probably because the tools and analysis methods to measure the social support were different.

We found that self-control’s direct effect on resilience was significant. In other words, self-control increases the level of resilience significantly, and as the level of self-control is high, the adolescent is capable of overcoming any danger or failure to which he/she is exposed.

The direct or indirect effect of self-control on problem behaviors was not significant, but the total effect was shown to be significant. This result corresponds to the previous studies [14,44] presenting the finding that self-control is a major factor explaining adolescents’ delinquency and deviation [14] and the finding that as the level of self-control is high, internalizing and externalizing problem behaviors are reduced [44]. Such findings suggest that improving adolescents’ self-control contributes to reducing problem behaviors and improving resilience. Thus, when intervention programs are developed for adolescents exposed to psychological maltreatment, it is important to reflect content that can improve self-control.

Finally, psychological maltreatment was a risk factor that affects adolescents’ problem behaviors significantly. In addition, adolescents’ self-control acted as a protective factor and decreased problem behaviors significantly. Such results indicate the importance of preventing adolescents’ psychological maltreatment. In order to prevent and manage adolescents’ psychological maltreatment, it is necessary to establish governmental policies and systems. Above all, parents, as the main rearers in each family, need to develop a proper perception of psychological maltreatment. To practice rearing appropriately, various education and promotion programs also need to be conducted. Throughout the society, the danger of child abuse needs to be made known continually. Particularly, campaigns and systematic education programs to prevent psychological maltreatment need to be conducted actively. In addition, there need to be programs to improve self-control, which is a psychological protective factor to reduce problem behaviors and improve resilience among adolescents. To this end, it is necessary to develop close relationships between adolescents and their parents as well as positive relationships with teachers and companions at school in consideration of the importance of sufficient social support. Since self-control is significantly affected by parents’ rearing behaviors, education needs to be practiced for both actual and potential rearers regarding proper rearing practices. Health education also needs to be conducted for adolescents themselves to improve their own psychological protective factors. Problem behaviors are a serious problem not only for adolescents themselves but also for community members who will take the lead of our society in the future. Therefore, there is an urgent need for individuals, families, and schools as well as the entire society all to put forth efforts to reduce adolescents’ problem behaviors.

This study establishes a structural model to analyze the effects of psychological maltreatment on adolescents’ problem behaviors. This study is of significance in that social psychological protective factors (social support and self-control) were found to be effective as a medium in reducing adolescents’ problem behaviors.

## 5. Conclusions

Child abuse is a serious social issue although the focus has been on physical abuse. In contrast, the danger of psychological maltreatment has been relatively scarcely recognized although it is a type of abuse mostly frequently committed. Its negative effect on children and adolescents also has been neglected despite its seriousness.

Findings of this study suggest that psychological maltreatment affects adolescents’ problem behaviors. It was also shown that psychological maltreatment also affects adolescents’ social protective factors (perceived social support) and personal psychological protective factors (self-control) negatively and that adolescents’ perceived social support and self-control affect resilience significantly. In other words, psychological maltreatment increases problem behaviors among adolescents and affects social psychological protective factors (social support and self-control) negatively.

In order to reduce adolescents’ problem behaviors, psychological maltreatment must therefore be eradicated. In addition, it is necessary to develop ways to strengthen social psychological protective factors among adolescents. Parents in each family need to practice appropriate rearing and to put forth efforts to induce positive parent–child interactions. Particularly in Korean society and in the families in it, the perception of psychological maltreatment needs to be changed. Specifically, the danger and significance of negative effects of psychological maltreatment on adolescents’ growth and development (problem behaviors, mental health deterioration, etc.) need to be made known actively through education programs.

The findings of this study may be utilized in interventions at clinical treatment settings for children and adolescents who experience maltreatment. These findings also may be used for practical guidelines to improve adolescents’ mental health in families, schools, and local communities. In addition, it is expected that this study will lead to more investigations and structural model studies on various factors that affect adolescents’ problem behaviors as well as psychological maltreatment. It is also hoped that systematic longitudinal research and qualitative research on the effects of psychological maltreatment on adolescents’ problem behaviors continue to be conducted.

The following are possible avenues for future research. In hypothetical model research, research is possible if the minimal sample size (200) suggested by the maximum-likelihood classification method is satisfied [27]. However, there are limitations in generalizing the present research results because the subjects were selected by convenience sampling. Therefore, it is necessary to repeat the present research targeting students in various regions.

## Figures and Tables

**Table 1:** Participants’ Characteristics.

		(N = 238)
Variables	Categories	n (%)
Gender	Male	124 (52.1)
	Female	114 (47.9)
School Type	Middle School	73 (30.7)
	Academic High School	120 (50.4)
	Vocational High School	45 (18.9)
Friendship	Good	204 (85.7)
	Usually	33 (13.9)
Academic Stress	High	41 (17.2)
	Usually	156 (65.5)
	Low	41 (17.2)
Family Structure	Two Parent	160 (67.2)
	One Parent	24 (10.1)
	Grandparent	7 (12.9)
	Others	47 (19.7)
Economic Status	High	67 (28.2)
	Middle	128 (53.8)
	Low	43 (18.1)
Paternal Education	Middle School or Less	9 (13.8)
	High School	95 (39.9)
	College	101 (42.4)
	Graduate School	21 (18.8)
Maternal Education	Middle School or Less	7 (12.9)
	High School	119 (50.0)
	College	95 (39.9)
	Graduate School	6 (12.5)
Paternal Job	Office Work	90 (37.8)
	Technical Production	72 (30.3)
	Sale Service	48 (20.2)
	Others	28 (11.8)
Maternal Job	Working	154 (64.7)
	Housewife	76 (31.9)
	No Answer	8 (03.4)

**Table 2:** Descriptive Statistics of Study Variable.

				(N = 238)
Variables (Item)	Range	Mean ± SD	Skewness	Kurtosis
Perceived Social Support (23)				
Family (8)	8~40	33.28 ± 6.21	−0.65	−0.07
Friend (8)	8~40	30.98 ± 5.20	−0.30	0.66
Teacher (7)	7~35	25.36 ± 4.72	0.02	−0.22
Resilience (27)				
Controllability (9)	9~45	31.67 ± 5.27	0.24	0.37
Affirmative (9)	9~45	33.74 ± 5.89	−0.24	0.54
Sociability (9)	9~45	33.78 ± 5.28	−0.33	1.03
Self-Control (19)				
Long-term Control (9)	9~45	33.81 ± 5.08	0.21	0.65
Short-term Control (10)	10~50	38.02 ± 5.27	−0.18	−0.38
Psychological Maltreatment (17)				
Emotional Abuse (9)	9~36	9.56 ± 1.12	2.31	5.63
Neglect (8)	8~32	8.34 ± 0.87	2.79	7.28
Problem Behavior (60)				
Internalizing (30)	30~90	8.97 ± 8.34	1.19	0.83
Externalizing (30)	30~90	6.68 ± 5.72	1.61	5.24

**Table 3:** Correlations among the Measured Variables.

(N = 238)												
	x^1^	x^2^	y^1^	y^2^	y^3^	y^4^	y^5^	y^6^	y^7^	y^8^	y^9^	y^10^
x^1^: Emotional abuse	1											
x^2^: Neglect	0.361 **	1										
y^1^: Family	−0.283 **	−0.181 **	1									
y^2^: Friend	−0.153 *	−0.094	0.487 **	1								
y^3^: Teacher	−0.170 **	−0.035	0.445 **	0.561 **	1							
y^4^: Controllability	−0.167 *	−0.032	0.430 **	0.553 **	0.442 **	1						
y^5^: Affirmative	−0.222 **	−0.048	0.529 **	0.570 **	0.487 **	0.663 **	1					
y^6^: Sociability	−0.109	0.035	0.418 **	0.638 **	0.447 **	0.665 **	0.662 **	1				
y^7^: Long-term	−0.142 *	−0.089	0.387 **	0.497 **	0.428 **	0.693 **	0.552 **	0.558 **	1			
y^8^: Short-term	−0.030	−0.031	0.163 *	0.375 **	0.304 **	0.486 **	0.415 **	0.382 **	0.538 **	1		
y^9^: Internalizing	0.312 **	0.20 **	−0.072	−0.286 **	−0.067	−0.230 **	−0.273 **	−0.173 **	−0.116	−0.333 **	1	
y^10^: Externalizing	0.231 **	0.189 **	−0.053	−0.224 **	−0.101	−0.284 **	−0.253 **	−0.165 *	−0.283 **	−0.471 **	0.662 **	1

* *p* < 0.05, ** *p* < 0.001, Psychological Maltreatment: x^1^ and x^2^; Perceived Social Support: y^1^, y^2^, and y^3^; Resilience: y^4^, y^5^, and y^6^; Self-Control: y^7^ and y^8^; Problem Behavior: y^9^ and y^10^.

**Table 4:** Standardized Estimations of the Measured Variables.

Measured Variables	RW ^1^	SE ^2^	CR ^3^	*p*	SRW ^4^	SMC ^5^
Perceived Social Support						
Friend	1				0.799	0.639
Family	0.964	0.101	9.556	<0.001	0.645	0.416
Teacher	0.768	0.076	10.041	<0.001	0.676	0.456
Resilience						
Affirmative	1				0.805	0.647
Controllability	0.931	0.066	14.200	<0.001	0.837	0.700
Sociability	0.891	0.066	13.423	<0.001	0.800	0.640
Self-Control						
Long-term Control	1				0.802	0.643
Short-term Control	0.868	0.088	9.902	<0.001	0.671	0.450
Psychological Maltreatment						
Emotional Abuse	1				0.726	0.526
Neglect	0.530	0.154	3.437	<0.001	0.497	0.247
Problem Behavior						
Internalizing	1				0.701	0.491
Externalizing	0.924	0.135	6.853	<0.001	0.945	0.892

^1^ Regression weight, ^2^ standard error, ^3^ critical ratio, ^4^ standard regression weight, and ^5^ squared multiple correlation.

**Table 5:** Model Fit of the Hypothetical Model.

Model	CMIN ( $x^{2}$)			GFI ^1^	AGFI ^2^	CFI ^3^	SRMR ^4^	RMSEA ^5^
	χ^2^	DF	*p*					
Reference			>0.05	≥0.9	≥0.8~9	≥0.9	≤0.08	0−0.10
Hypothetical	151.62	44	<0.001	0.908	0.836	0.911	0.06	0.10

^1^ Goodness of fit index, ^2^ adjusted goodness of fit index, ^3^ comparative fit index, ^4^ standardized root mean square residual, and ^5^ root mean square error of approximation.

**Table 6:** Standardized Estimations of the Hypothetical Model.

Endogenous Variables	RW ^1^	SE ^2^	CR ^3^	*p*	SRW ^4^	SMC ^5^
Exogenous Variables						
Perceived Social Support						0.115
Psychological Maltreatment	−1.733	0.628	−2.759	0.006	−0.339	
Resilience						0.899
Psychological Maltreatment	0.251	0.381	0.659	0.510	0.043	
Perceived Social Support	0.578	0.131	4.406	<0.001	0.507	
Self-Control	0.612	0.134	4.579	<0.001	0.526	
Self-Control						0.532
Psychological Maltreatment	0.341	0.484	0.706	0.480	0.068	
Perceived Social Support	0.735	0.095	7.721	<0.001	0.750	
Problem Behavior						0.397
Psychological Maltreatment	2.691	1.073	2.509	0.012	0.374	
Perceived Social Support	0.354	0.415	0.853	0.394	0.252	
Resilience	0.392	0.641	0.611	0.541	0.318	
Self-Control	−1.249	0.566	−2.206	0.027	−0.871	

^1^ Regression weight, ^2^ standard error, ^3^ critical ratio, ^4^ standard regression weight, and ^5^ squared multiple correlation.

**Table 7:** Direct, Indirect, and Total Effect of the Hypothetical Model.

Endogenous Variables	Standardized Direct Effect (*p*)	Standardized Indirect Effect (*p*)	Standardized Total Effect (*p*)
Exogenous Variables			
Perceived Social Support			
Psychological Maltreatment	−0.339 (0.047) *		−0.339 (0.047) *
Resilience			
Psychological Maltreatment	0.043 (0.854)	−0.269 (0.054)	−0.226 (0.102)
Perceived Social Support	0.507 (0.023) *	0.394 (0.002) *	0.901 (0.011) *
Self-Control	0.526 (0.004) *		0.526 (0.004) *
Self-Control			
Psychological Maltreatment	0.068 (0.392)	−0.254 (0.037) *	−0.186 (0.120)
Perceived Social Support	0.750 (0.003) *		0.750 (0.003) *
Problem Behavior			
Psychological Maltreatment	0.374 (0.134)	0.005 (0.964)	0.379 (0.024) *
Perceived Social Support	0.252 (0.579)	−0.366 (0.311)	−0.115 (0.221)
Resilience	0.318 (0.442)		0.318 (0.442)
Self-Control	−0.871 (0.121)	0.167 (0.397)	−0.703 (0.043) *

* *p* < 0.05

## Data Availability

The data used is confidential, and the study participants have not consented to data sharing. Due to the sensitive nature of the personal information in the questions asked in this study, the survey respondents were assured that the raw data would be kept confidential and would not be shared.
